# Supplementary material for: Dingoes, companions in life and death: The significance of archaeological canid burial practices in Australia
Source: PLoS One. 2023 Oct 20;18(10):e0286576. doi: 10.1371/journal.pone.0286576 (PMC10588905; doi:10.1371/journal.pone.0286576)
Supplement: Attachment — Submitted filename: Response to reviewers.docx [file pone.0286576.s001.docx]

**Response to Reviewers and Academic Editor**

**Reviewer #1**

We thank the reviewer for their kind and constructive feedback. We have addressed their points as follows (pages and lines refer to original MS submission, not revised one):

- Page 3, Lines 51-53: We agree with this statement in principle. However, with respect to this specific instance: as dingoes are very thousands of kilometres removed from the biogeographic source of their wild ancestor (an East Asian wolf) it seems reasonable to assume that their ancestors were transported in a domesticated state. The only alternative explanation is a series of stepwise maritime translocations of wild dog stock throughout Mainland and Island Southeast Asia, but there is no archaeological or palaeontological evidence that populations of such animals existed in antiquity - aside from in New Guinea but for which the same issue of distance of distance from the ancestral home range exists. We have edited this section to better reflect the above contentions.
- Page 6, Line 132: Spelling error corrected
- Page 11, Line 293: Grammatical error corrected
- Page 14, Line 313: Grammatical/spelling errors corrected

**Reviewer #2**

We thank the reviewer for their kind and constructive feedback. We have addressed their points as follows (pages and lines refer to original MS submission, not revised one):

- Line 14: The reviewer’s comment that Jackson et al. in both instances argue for *Canis familiaris* is correct. We have modified this paragraph to reflect the debatable taxonomy of dingoes.
- Line 51: We have added reference to these in text; ultimately this source (Abbott) was already who the cited papers’ arguments mostly rely on, but we have now cited his works directly in text along with Fairfax 2019.
- Line 88: The idea of tamed vs untamed/domestic vs feral sensu Ballard and Wilson relies on a lack of definitive evidence for dingoes being domesticated in the past, hence “tamed” and “untamed” being safer designations. However, this contention is based on the interpretation of the biology/genetics/behaviour of modern wild dingoes which have experienced natural selection for millennia and thus may have lost many indicators of domestic heritage (whilst others remain such as gross cranial morphology). We have added some text to reflect this contention.
- Lines 525-542: We agree that the debate over the taxonomic status of dingoes involves both discussions of domesticity as well as morphological/genetic/behavioural differentiation, but ultimately those opinions on domesticity are derived from observations of morphology, genetic and behavioural characters. We have added another paragraph to convey this.

**Academic Editor**

Our manuscript has been edited and formatted to address the points raised by the reviewers. In addition, we have addressed your requests regarding formatting of the text and headings, figure and table captions, their in-text citations, and corresponding file names. We have also included within the acknowledgements section a statement concerning the obtainment of permits to carry out the research.
